# Characteristics and outcomes of neurosurgical patients in an emergency hospital admission setting

**DOI:** 10.3389/fneur.2025.1516229

**Published:** 2025-05-29

**Authors:** Yuehua Li, Wei Chen

**Affiliations:** ^1^Operating Room, Department of Anesthesiology, West China Hospital, Sichuan University, Chengdu, China; ^2^West China School of Nursing, Sichuan University, Chengdu, China; ^3^Department of Neurosurgery, West China Hospital, Sichuan University, Chengdu, China

**Keywords:** neurosurgical patients, characteristics, emergency, admission, outcome

## Abstract

**Objective:**

This study aims to investigate the characteristics of patients admitted to the neurosurgery department in an emergency manner.

**Methods:**

A population-based, retrospective cohort study including consecutive patients admitted to the department of neurosurgery as emergency admissions in a non-profit tertiary care university hospital was conducted. Data on demographic information, clinical characteristics and outcomes were collected and evaluated. Patients were stratified into five disease groups (vascular disease, trauma, oncology, spine, and others) according to their main diagnoses at the time of admission.

**Results:**

A total of 4,149 cases (median age 52 years, 54.5% male) were included in this study. Vascular disease was the most common reason for emergency admission (73.5%). Significant differences were found among the five disease groups in sex (*P* < 0.001), age (*P* < 0.001), surgery (*P* < 0.001), and season (*P* = 0.009) but not in the length of stay (*P* = 0.784). Multivariate logistic regression analysis identified male sex, older age, short length of stay, surgery not performed, and disease type (particularly trauma) as independently associated with in-hospital mortality.

**Conclusions:**

There is demographic heterogeneity and clinical differences exist among neurosurgical patients admitted to the neurosurgery department as an emergency. In addition, male sex, older age, shorter length of stay, absence of surgery, and admission due to trauma emerged as independent predictors of higher in-hospital mortality.

## Introduction

The Lancet Commission on Global Surgery emphasized the global need for surgical care and the gap existing between surgical need and the equitable provision of safe surgical care (1). The neurosurgical community is responsible for identifying areas with major current gaps and outlining strategies for interventions (2). In this context, it is necessary to obtain an adequate understanding of the characteristics of neurosurgical patients prior to making health care policies.

Neurosurgical patients are admitted to hospital via two main mechanisms: outpatient admissions and emergency department admissions. The former is available for most patients whose condition is relatively stable, and these patients usually need to wait a particular period; whereas the latter is reserved for patients who require hospitalization immediately without a wait. It is common for neurosurgical patients to require admission to hospitalization in an emergency manner in clinical practice. According to the 2017 almanac of the West China Hospital, Sichuan University, 2,284 patients were admitted to the neurosurgery department as emergency admissions, accounting for roughly 7.5% of all emergency admissions and nearly one-quarter of all neurosurgery admissions. The features of hospitalized patients have been extensively described in published studies aimed at investigating a variety of aspects (such as patient diagnosis, treatment, and prognosis) of neurological diseases. However, data on the characteristics of neurosurgical patients who are admitted through emergency manner are lacking. Thus, this study aims to address the knowledge gap regarding the overall characteristics and outcomes of patients admitted to the neurosurgery department via emergency admission. By identifying common demographic and clinical factors across diagnostic categories, we aim to provide evidence that can inform clinical decision-making and health policy development for emergency neurosurgical services. However, the primary objective of this study was to provide a system-level overview of neurosurgical patients admitted via emergency services, focusing on demographics, diagnostic categories, treatment patterns, seasonal trends, and in-hospital mortality as the most reliably available and clinically relevant outcome in our large-scale retrospective dataset.

## Methods

### Study design

We conducted a population-based retrospective cohort study that included consecutive patients who were admitted to the neurosurgery department in an emergency manner during a 2-year period from January 1, 2017 to December 31, 2018 at West China Hospital, Sichuan University. Specifically, the date of entrance into this study for each patient was within the above period and the same as the date of admission to the neurosurgery department, and the date of exit from this study was the actual date that patient was discharged. The West China Hospital, Sichuan University is a 4,300-bed non-profit, tertiary care, national comprehensive regional transfer center in China, and its health care area covers the southwest area and nearly one-fifth of the population (300 million) of China (3). The neurosurgery department of the hospital ranked #3 among specialized centers in China. Under the health-care system in China (4, 5), patients visit our hospital spontaneously or via the medical referral system. This study was approved by Biomedical Research Ethics Committee of West China Hospital, Sichuan University. Informed consent was waived for this retrospective analysis of deidentified data.

### Study population, definitions, and data collection

Patients were included in this study if they were admitted to the neurosurgery department as an emergency admission. Patients admitted to the neurosurgery department for reasons unrelated to cranial or spinal conditions (e.g., severe pneumonia with a history of craniotomy 3 weeks ago) were included. We also excluded patients who had no available or incomplete information (e.g., age or diagnosis) regarding admission, hospitalization, or discharge during the study period (*n* = 61). Hospital records of patients were reviewed. Demographic information and clinical characteristics were retrieved and included the patient sex, age, month of admission, length of stay, operative data, and outcomes at the time of discharge. Operative data included whether and when neurosurgical operations (not including gamma knife radiosurgery and cerebral digital subtraction angiography) were performed during the hospitalization. For the purposes of this study, patients were categorized into the following five groups, namely, vascular disease, trauma, oncology, spine, and others, according to their main diagnoses (International Classification of Disease, Tenth Revision) at the time of admission. Comprehensive diagnostic criteria for each group, including specific conditions allocated to the “Others” category (such as postoperative infections following clipping or tumor surgery), are provided in detail in the Supplementary material. To investigate the seasonal pattern of the study population, we categorized the patients into four groups: those seen in spring, summer, autumn, and winter. The primary outcome of interest was in-hospital mortality, which was abstracted from the electronic medical records of patients.

### Statistical methods

Categorical variables are expressed as counts with percentages, and continuous variables as medians and interquartile ranges (IQR). We compared the basic demographics and clinical characteristics among the five disease groups using the Chi-squared test or Fisher's exact test for categorical variables and Student's *t*-test or Mann–Whitney *U* test for continuous variables in a univariate analysis. We performed a multivariate logistic regression analysis to determine which variables are independently associated with in-hospital mortality, with results presented as odds ratios (ORs) and 95% confidence intervals (CIs). Given the different etiologies in the disease groups and the effects of factors such as age and sex, which might differ between disease groups, interaction, and stratified analyses considering disease type and other factors in the mortality analysis were performed. All statistical analyses were performed using SPSS (version 27.0) and the statistical package of R (version 3.1.2). The statistical significance level was determined by a 2-tailed *P* < 0.05 for all analyses.

## Results

We included 4,149 cases during the study period. The median age was 52 years (IQR 44–62 years). There were 2,263 males (54.5%) and 1,886 females (45.5%). A total of 2,489 cases (60.0%) underwent surgery at a median time of 3 days after admission, and 951 cases (22.9%) underwent surgery within 24 h after admission. The median length of stay was 12 days (IQR 9.0–18.0 days). The characteristics of the study population were stratified by the type of disease and are summarized in Table 1.

**Table 1:** Characteristics of the study population stratified by type of disease.

**Characteristic**	**Total (*n* = 4,149)**	**Vascular (*n* = 3,050)**	**Trauma (*n* = 336)**	**Oncology (*n* = 449)**	**Spine (*n* = 77)**	**Others (*n* = 237)**	***P*-value**
**Sex**							
Female	1,886 (45.5%)	1,467 (48.1%)	96 (28.6%)	196 (43.7%)	26 (33.8%)	101 (42.6%)	< 0.001
Male	2,263 (54.5%)	1,583 (51.9%)	240 (71.4%)	253 (56.3%)	51 (66.2%)	136 (57.4%)	
Age, years	52.0 (44.0–62.0)	52.0 (44.0–62.0)	47.0 (25.3–63.0)	29.0 (9.0–49.0)	36.5 (14.5–50.8)	17.5 (2.0–47.0)	< 0.001
Length of stay, days	12.0 (9.0–18.0)	12.0 (9.0–18.0)	10.5 (6.0–22.0)	10 (7.0–15.0)	11.0 (8.0–16.3)	9.0 (5.0–16.0)	0.784
**Surgery**							
Yes	2,489 (60.0%)	1,702 (55.8%)	180 (53.6%)	393 (87.5%)	58 (75.3%)	156 (65.8%)	< 0.001
No	1,660 (40.0%)	1,348 (44.2%)	156 (46.4%)	56 (12.5%)	19 (24.7%)	81 (34.2%)	
Timing of surgery, days (*n* = 2,489)	3.0 (1.0–5.0)	3.0 (1.0–5.0)	1.0 (0.0–1.0)	3.0 (2.0–5.0)	4.0 (1.0–6.0)	2.0 (1.0–4.0)	< 0.001
**Season**							
Spring	1,099 (26.5%)	842 (27.6%)	71 (21.1%)	120 (26.7%)	16 (20.8%)	50 (21.1%)	0.009
Summer	937 (22.6%)	652 (21.4%)	91 (27.1%)	103 (22.9%)	20 (26.0%)	71 (30.0%)	
Autumn	1,079 (26.0%)	771 (25.3%)	100 (29.8%)	119 (26.5%)	24 (31.2%)	65 (27.4%)	
Winter	1,034 (24.9%)	785 (25.7%)	74 (22.0%)	107 (23.8%)	17 (22.1%)	51 (21.5%)	
**Outcome**							
Survival	3,948 (95.2%)	2,908 (95.3%)	294 (87.5%)	437 (97.3%)	76 (98.7%)	233 (98.3%)	< 0.001
Death	201 (4.8%)	142 (4.7%)	42 (12.5%)	12 (2.7%)	1 (1.3%)	4 (1.7%)	

Sex, surgery, season, and outcome are expressed as counts with percentages; age, length of stay, and the timing of surgery (time to surgery from admission) are presented as medians and interquartile ranges.

**Figure 1:**
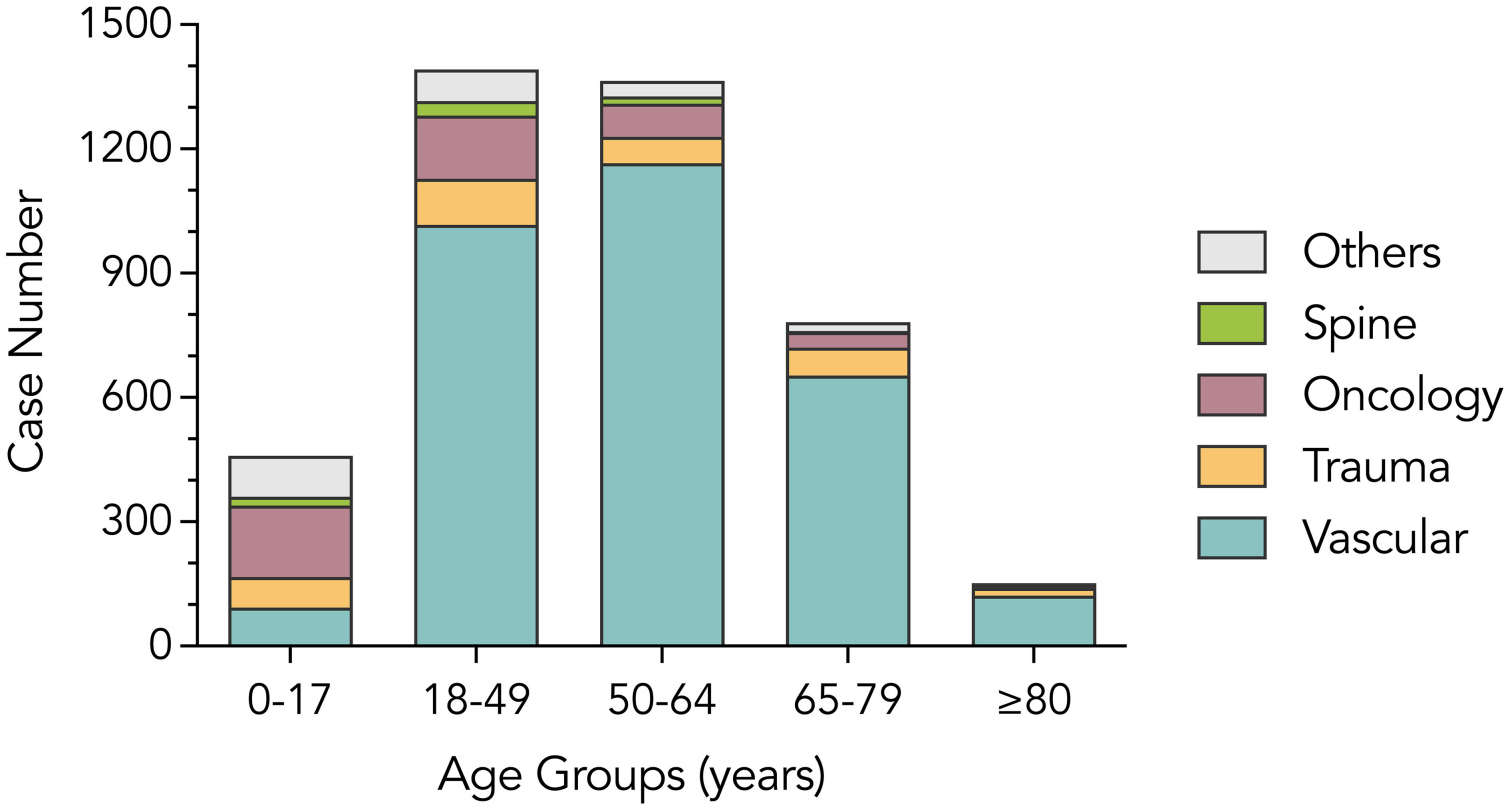
Age profile of the study population.

In this study, vascular disease was the most common type of emergency admission (3,050 cases, 73.5%), followed by neuro-oncological diseases (449 cases, 10.8%) and trauma (336 cases, 8.1%). Univariable analysis results revealed that there were statistically significant differences in sex (*P* < 0.001), age (*P* < 0.001), surgery (*P* < 0.001), and season (*P* = 0.009), while no significant difference was observed in the length of stay (*P* = 0.784) among the five disease groups, as shown in Table 1. Patients in the vascular disease group were older than those in the other four groups. In contrast, patients in the trauma group appeared to have undergone surgery early and had higher in-hospital mortality. With regard to the seasonality of diseases, fewer patients were admitted to the hospital in summer overall (22.6%) than in other seasons, especially in the vascular disease group (21.4%). However, this pattern was not consistent across other disease groups. The age distribution across disease categories is illustrated in Figure 1, showing that vascular cases were predominantly concentrated in older age groups, while trauma cases were more frequent among younger patients.

All-cause in-hospital death occurred in 201 (4.8%) patients. The multivariate logistic regression analysis showed that a male sex (OR: 1.460; 95% CI: 1.077–1.978; *P* = 0.015), age (OR: 1.025; 95% CI: 1.016–1.033; *P* < 0.001), hospital days (OR, 0.980; 95% CI: 0.965–0.996; *P* = 0.012), without surgery (OR: 1.445; 95% CI: 1.052–1.985; *P* = 0.023), and disease type (*P* < 0.001) were independently associated with higher in-hospital mortality, but season was not (*P* = 0.323), as presented in Table 2. A subgroup analysis of interactions between disease type and potential factors for in-hospital death was not statistically significant (*P* values of all interaction test were >0.05), as shown in Table 3.

**Table 2:** Multivariable binomial logistic regression analysis for in-hospital mortality.

**Variable**	**OR**	**95% CI**		***P*-value**
Male sex	1.460	1.077	1.978	0.015
Age	1.025	1.016	1.033	< 0.001
Length of stay	0.980	0.965	0.996	0.012
Without surgery	1.445	1.052	1.985	0.023
**Season**				0.323
Winter (ref)	1.000			
Spring	0.771	0.508	1.169	0.220
Summer	1.133	0.760	1.689	0.539
Autumn	0.905	0.606	1.349	0.623
**Disease type**				< 0.001
Vascular (ref)	1.000			
Trauma	3.445	2.354	5.042	< 0.001
Oncology	0.979	0.527	1.822	0.948
Others	0.586	0.212	1.621	0.303
Spine	0.465	0.063	3.406	0.451

CI, confidence interval; OR, odds ratio.

**Table 3:** Association between disease type and in-hospital mortality, stratified by potential factors.

**Subgroup**	**In-hospital death**, ***n*** **(%)**		**Unadjusted**		**Adjusted** ^*^	
	**No**	**Yes**	**OR (95% CI)**	***P*** **-value for interaction**	**OR (95% CI)**	***P*** **-value for interaction**
**Sex**						
Female	2,134 (54.1%)	129 (64.2%)	0.828 (0.687–0.998)	0.696	0.918 (0.713–1.182)	0.987
Male	1,814 (45.9%)	72 (35.8%)	0.882 (0.687–1.131)				0.920 (0.760–1.115)	
**Age, years**						
< 65	3,100 (78.5%)	115 (57.2%)	0.953 (0.809–1.123)	0.269	0.945 (0.789–1.118)	0.324
≥65	848 (21.5%)	86 (42.8%)	0.768 (0.532–1.110)				0.774 (0.529–1.131)	
**Length of stay, days**						
< 10	1,918 (48.6%)	137 (68.2%)	0.797 (0.656–0.969)	0.230	0.842 (0.685–1.034)	0.395
≥10	2,030 (51.4%)	64 (31.8%)	0.961 (0.763–1.211)				0.964 (0.764–1.216)	
**Surgery**						
Yes	1,545 (39.1%)	115 (57.2%)	0.753 (0.572–0.992)	0.078	0.962 (0.797–1.162)	0.231
No	2,403 (60.9%)	86 (42.8%)	1.000 (0.835–1.199)				0.785 (0.589–1.046)	
**Season**						
Spring	1,056 (26.7%)	43 (21.4%)	0.860 (0.612–1.210)	0.281	0.915 (0.635–1.317)	0.219
Summer	884 (22.4%)	53 (26.4%)	0.783 (0.584–1.050)				0.840 (0.629–1.127)	
Autumn	1,028 (26.0%)	51 (25.4%)	0.711 (0.501–1.010)				0.744 (0.517–1.069)	
Winter	980 (24.8%)	54 (26.9%)	1.044 (0.817–1.334)				1.148 (0.888–1.484)	

^*^Each stratification adjusted for all the factors (sex, age, length of stay, surgery, and season) except for the stratification factor itself.

CI, confidence interval; OR, odds ratio.

## Discussion

In the context of limited evidence describing the characteristics of neurosurgical patients admitted via emergency services, this study was conducted to provide a comprehensive system-level overview of this population. Our findings demonstrate substantial demographic and clinical heterogeneity across diagnostic groups. Furthermore, several factors—including male sex, older age, shorter length of stay, absence of surgical intervention, and disease type (particularly trauma)—were independently associated with higher in-hospital mortality.

The present study indicates that there were sex differences among the five groups. The proportion of male patients was higher than that of female patients in the study population (1.2:1.0), and this difference was much more obvious in the trauma group (2.5:1.0). This finding is consistent with the findings of previous studies that have reported a male predominance among traumatic brain injuries (6–8), with male-to-female ratios ranging from 1.2:1.0 (9) to 4.6:1.0 (10). The predominance of male patients in the trauma group aligns with prior epidemiological studies that attribute this trend to higher exposure to risk-prone activities, occupational hazards, and behavioral factors among males. For example, Peeters et al. and Blennow et al. (7, 8) reported male-to-female ratios ranging from 1.2:1 to 4.6:1 in TBI cases. Beyond environmental exposure, biological sex differences in inflammatory response, hormonal modulation, and neuroplasticity may also contribute to sex-based outcome disparities after brain injury. In addition, data from the WHO Mortality Database show that brain and central nervous system cancer mortality rates are ~1.5 times higher in males than in females (11). Age-related differences among this population are also significant. In the present study, patients with vascular diseases were older than those in other disease groups. This finding has been confirmed in past cohort studies in which the mean ages of trauma patients ranged from 22 to 49 years (10, 12), while that of vascular diseases (mainly including subarachnoid hemorrhage and intracerebral hemorrhage) was 55–69 years (13, 14). Although vascular and trauma-related admissions constituted the majority of cases, it is important to highlight key observations from the neuro-oncology and spine groups. Patients with neuro-oncological conditions had a relatively younger median age and high surgical intervention rates, consistent with the urgent need for decompression or mass effect relief in selected tumor cases. Conversely, spinal cases, while less frequent, also showed a high proportion of surgical treatment and a low in-hospital mortality rate, possibly reflecting effective triage and favorable surgical indications. These findings suggest that neuro-oncological and spine emergencies, although smaller in volume, contribute meaningfully to the overall clinical spectrum and should be further explored in disease-specific studies.

We did not find any significant differences in length of stay among the five groups, but an association between length of stay and in-hospital mortality was observed; in-hospital mortality was increased among patients with a relatively short length of stay. This finding suggests that most deaths of neurosurgical patients who admitted to the hospital in an emergency manner occurred in the early stage of hospitalization. Therefore, it seems necessary and worthwhile to increase efforts in early recognition and aggressive intervention in such patients. Moreover, there were statistically significant differences between patients with and without surgery and across different timings of surgery among the five groups. More than half of the patients underwent neurosurgical surgery, and this proportion was especially high among patients with trauma and vascular diseases. In a meta-analysis of the treatment of intracerebral and intraventricular hemorrhage, Scaggiante et al. (15) demonstrated that select patients with supratentorial intracerebral hemorrhage benefit more from minimally invasive surgery than from other treatments. The in-hospital mortality was significantly lower in patients with surgery than in those without, demonstrating the potential role of surgical treatment in the management of emergency patients. However, it is important to note that the low level of mortality among patients who undergo surgery may not simply be due to the effectiveness of surgery, and vice versa. The role of surgical interventions still needs to be investigated, especially in patients with spontaneous intracerebral hemorrhage (16). Although our data showed an association between surgical intervention and lower in-hospital mortality, this finding should not be interpreted as evidence that surgery is uniformly required or beneficial in all neurosurgical emergency cases. Many patients present with conditions so severe that even the most aggressive treatment is unlikely to alter the outcome. Clinical decision-making must be individualized, incorporating not only imaging and physiological data, but also patient-specific factors such as age, comorbidities, and prognosis. Therefore, the observed mortality benefit among surgical patients likely reflects both selection bias and the presence of clinical scenarios more amenable to intervention.

The seasonal pattern of admissions differed significantly between the disease groups. It is important to note that we conducted this analysis with the overall data. The available published study mainly focused on the seasonality of stroke in the population. Using data from a worldwide multicentre trial, Herweh et al. found that climatic and seasonal conditions were associated with hypertensive intracerebral hemorrhage (17). Similar findings were also reported in another previous study conducted by Han et al. (18), who showed that there are distinct patterns of seasonal and monthly variation in the incidence of stroke. Although the exact mechanisms underlying these phenomena remain unclear, the seasonal patterns of diseases may need to receive more attention by health providers to more optimally allocate medical resources. In contrast, an association between seasonality and in-hospital mortality was not observed in the multivariate logistic regression analysis in the present study. Seasonal variation in neurosurgical emergencies, particularly vascular pathologies such as intracranial aneurysm rupture and traumatic brain injury, has been reported in previous studies. For instance, Herweh et al. (17) found associations between hypertensive intracerebral hemorrhage and colder seasons. In our study, we observed a lower number of vascular admissions during the summer, which may reflect seasonal blood pressure fluctuation or behavioral patterns. In terms of trauma, some mechanisms of injury—such as falls or traffic accidents—may increase during warmer months due to increased outdoor activity. While our current dataset does not include detailed mechanism-specific trauma categorization, future studies with more granular trauma data may help clarify such trends and guide seasonal resource allocation in emergency neurosurgical services.

We investigated the relationship between sex and in-hospital mortality and found that the risk of in-hospital mortality was higher in male than in female patients. Additionally, it has been shown that older age tends to be a predictor of increased in-hospital mortality. In a study of 2,212 patients with intracerebral hemorrhage, Marini et al. (19) found that males were independently associated with higher 90-day mortality and 1-year mortality rates. A possible explanation is that the burden of vascular risk factors and cardiovascular diseases is higher in men than in women (20). Evidence from a prior study of sex differences in patients with delayed cerebral ischaemia after subarachnoid hemorrhage suggested that sex and age interact to influence the risk of subarachnoid hemorrhage, probably through a sex-specific hormonal factor (21). Furthermore, one hypothesis is that sex-related differences in the response to brain injury may also play a role (22). The other predictors identified in the present study included shorter length of stay, with or without surgery, and disease type (particularly trauma). In previous studies, predictors such as Glasgow Coma Scale scores, age, hematoma volume and location, and the presence and amount of intraventricular hemorrhage have also been associated with outcomes (16, 23, 24). In another related paper, the authors reported that the predictors associated with severe brain injury included age, surgery, mechanism of trauma, subdural hemorrhage, and diffuse axonal injury (25). These findings, along with our results, demonstrate that some of the predictors of higher in-hospital mortality represent potential intervention targets. In interpreting the generalized predictors identified in this study, readers must recognize the inherent heterogeneity among disease groups. While our integrative approach highlights risk factors broadly relevant to emergency neurosurgical admissions, it does not substitute for detailed disease-specific analyses. Future studies focusing explicitly on prognostic factors unique to each neurosurgical condition are clearly warranted.

Our study has several limitations and should be interpreted with caution. First, we did not have access to full clinical data for this study. The absence of detailed clinical severity scores (e.g., GCS), perioperative complications, and functional outcome metrics (e.g., modified Rankin Scale) limits our ability to perform case-level clinical prognostication. These data were either inconsistently documented or unavailable within our retrospective registry. Future prospective studies designed with standardized data capture for severity and functional outcomes are warranted. Second, the disease groups were classified according to chief complaints and main diagnoses at the time of admission. It is possible that these could have changed during hospitalization. Third, this study was limited by its retrospective and single-center design. It is important to note that the characteristics of neurosurgical patients may vary around the world due to different underlying health care settings. These country-specific features, along with the specific regional population studied, may preclude the generalizability of the present results and their extension to other populations. Hence, in the future, well-designed global studies are encouraged to evaluate the characteristics of emergency neurosurgical patients to develop more optimal management strategies and improve patient outcomes.

Nevertheless, our study has strengths that are worth highlighting. This study is likely the first and largest series to study the characteristics and admission patterns of patients admitted to the neurosurgery department as an emergency. A potential significant clinical implication of these findings is that health care providers could be assisted in shifting tasks and clinical resources so that they are received by those patients who are most likely to benefit from particular intervention approaches. For example, establishing training programs for neurosurgeons and promoting the allocation of medical resources to treating trauma patients admitted as emergencies may improve the high mortality observed in this patient group.

## Conclusions

In conclusion, we identified significant demographic and clinical differences among neurosurgical patients admitted to the neurosurgery department as an emergency. In addition, male sex, older age, shorter length of stay, absence of surgery, and admission due to trauma emerged as independent predictors of higher in-hospital mortality. These findings may assist health care providers in shifting tasks and clinical resources toward those patients who may benefit from particular intervention approaches.

## Data Availability

The data analyzed in this study is subject to the following licenses/restrictions: Data are available from the corresponding author upon reasonable request. Requests to access these datasets should be directed to WC, chenwei0068@wchscu.cn.
