# Supplementary material for: Characteristics and outcomes of neurosurgical patients in an emergency hospital admission setting
Source: Front Neurol. 2025 May 29;16:1516229. doi: 10.3389/fneur.2025.1516229 (PMC12158670; doi:10.3389/fneur.2025.1516229)
Supplement: Supplementary file 1 [file Data_Sheet_1.DOCX]

**Supplementary Information**

Title: Characteristics and outcomes of neurosurgical patients in an emergency hospital admission setting

**Supplemental Methods**

Detail definitions of five disease groups (vascular disease, trauma, oncology, spine and others), according to the International Classification of Disease, Tenth Revision, ICD-10.

Patients with the following diagnosis were categorized into the **vascular disease** group:

D18.0 Hemangioma, any site (intracranial)

G45.9 Transient cerebral ischemic attack, unspecified

G81.9 Hemiplegia, unspecified (caused by intracerebral haemorrhage)

I60.0 Subarachnoid haemorrhage from carotid siphon and bifurcation

I60.1 Subarachnoid haemorrhage from middle cerebral artery

I60.2 Subarachnoid haemorrhage from anterior communicating artery

I60.3 Subarachnoid haemorrhage from posterior communicating artery

I60.4 Subarachnoid haemorrhage from basilar artery

I60.5 Subarachnoid haemorrhage from vertebral artery

I60.6 Subarachnoid haemorrhage from other intracranial arteries

I60.7 Subarachnoid haemorrhage from intracranial artery, unspecified

I60.8 Other subarachnoid haemorrhage

I60.9 Subarachnoid haemorrhage, unspecified

I61 Intracerebral haemorrhage

I61.0 Intracerebral haemorrhage in hemisphere

I61.1 Intracerebral haemorrhage in hemisphere

I61.2 Intracerebral haemorrhage in hemisphere, unspecified

I61.3 Intracerebral haemorrhage in brain stem

I61.4 Intracerebral haemorrhage in cerebellum

I61.5 Intracerebral haemorrhage, intraventricular

I61.6 Intracerebral haemorrhage, multiple localized

I61.9 Intracerebral haemorrhage, unspecified

I62 Other nontraumatic intracranial haemorrhage

I62.0 Subdural haemorrhage (acute)(nontraumatic)

I62.1 Nontraumatic extradural haemorrhage, Nontraumatic epidural haemorrhage

I62.9 Intracranial haemorrhage (nontraumatic), unspecified

I63 Cerebral infarction

I63.4 Cerebral infarction due to embolism of cerebral arteries

I63.5 Cerebral infarction due to unspecified occlusion or stenosis of cerebral arteries

I63.9 Cerebral infarction, unspecified

I65 Occlusion and stenosis of precerebral arteries, not resulting in cerebral infarction

I65.2 Occlusion and stenosis of carotid artery

I65.3 Occlusion and stenosis of multiple and bilateral precerebral arteries

I67 Other cerebrovascular diseases

I67.0 Dissection of cerebral arteries, nonruptured

I67.1 Cerebral aneurysm, nonruptured

I67.5 Moyamoya disease

I67.6 Nonpyogenic thrombosis of intracranial venous system

I72 Other aneurysm and dissection

I72.0 Aneurysm and dissection of carotid artery

I72.8 Aneurysm and dissection of other specified arteries

I77 Other disorders of arteries and arterioles

I77.0 Arteriovenous fistula, acquired

O99.4 Diseases of the circulatory system complicating pregnancy, childbirth and the puerperium

Q27.8 Other specified congenital malformations of peripheral vascular system

Q28 Other congenital malformations of circulatory system

Q28.0 Arteriovenous malformation of precerebral vessels

Q28.1 Other malformations of precerebral vessels

Q28.2 Arteriovenous malformation of cerebral vessels

Q28.3 Other malformations of cerebral vessels

Patients with the following diagnosis were categorized into the **trauma** group:

P10.1 Cerebral haemorrhage due to birth injury

P10.9 Unspecified intracranial laceration and haemorrhage due to birth injury

P12.0 Cephalhaematoma due to birth injury

S00.0 Superficial injury of scalp

S01.0 Open wound of scalp

S02.0 Fracture of vault of skull

S02.1 Fracture of base of skull

S06.0 Concussion

S06.2 Diffuse brain injury

S06.3 Focal brain injury

S06.4 Epidural haemorrhage, Extradural haemorrhage (traumatic)

S06.5 Traumatic subdural haemorrhage

S06.6 Traumatic subarachnoid haemorrhage

S06.7 Intracranial injury with prolonged coma

S06.8 Other intracranial injuries, Traumatic haemorrhage: cerebellar and intracranial NOS

S06.9 Intracranial injury, unspecified

S08.0 Avulsion of scalp

T90.5 Sequelae of intracranial injury

Patients with the following diagnosis were categorized into the **tumor** group:

C70.0 Cerebral meninges

C70.1 Spinal meninges

C70.9 Meninges, unspecified

C71.0 Cerebrum, except lobes and ventricles (Supratentorial NOS)

C71.1 Frontal lobe

C71.2 Temporal lobe

C71.3 Parietal lobe

C71.4 Occipital lobe

C71.5 Cerebral ventricle

C71.6 Cerebellum

C71.7 Brain stem (Fourth ventricle, Infratentorial NOS)

C71.8 Overlapping lesion of brain

C71.9 Brain, unspecified

C75.1 Pituitary gland

C75.3 Pineal gland

D33 Benign neoplasm of brain and other parts of central nervous system

D32.0 Cerebral meninges

D33.0 Brain, supratentorial

D33.1 Brain, infratentorial

D33.2 Brain, unspecified

D33.3 Cranial nerves, Olfactory bulb

D35 Benign neoplasm of other and unspecified endocrine glands

D35.2 Pituitary gland

D35.4 Pineal gland

D36.1 Peripheral nerves and autonomic nervous system

D43.0 Brain, supratentorial

D43.1 Brain, infratentorial

D43.2 Brain, unspecified

D44 Neoplasm of uncertain or unknown behaviour of endocrine glands

D44.3 Pituitary gland

D44.4 Craniopharyngeal duct

D44.5 Pineal gland

E23.6 Other disorders of pituitary gland

E34.8 Other specified endocrine disorders

G93.0 Cerebral cysts

G93.4 Encephalopathy, unspecified

G93.8 Other specified disorders of brain

G93.9 Disorder of brain, unspecified

R22.0 Localized swelling, mass and lump, head

R90.0 Intracranial space-occupying lesion

Patients with the following diagnosis were categorized into the **spinal** group:

C72.0 Spinal cord

C72.9 Central nervous system, unspecified

D32.1 Spinal meninges

D43.4 Spinal cord (Neoplasms of uncertain or unknown behaviour)

D48 Neoplasm of uncertain or unknown behaviour of other and unspecified sites

D48.0 Bone and articular cartilage

G82.2 Paraplegia, unspecified

G95.1 Vascular myelopathies

G95.8 Other specified diseases of spinal cord

G95.9 Disease of spinal cord, unspecified

Q05.7 Lumbar spina bifida without hydrocephalus

Q05.8 Sacral spina bifida without hydrocephalus

Q06.8 Other specified congenital malformations of spinal cord

Q07.0 Arnold-Chiari syndrome

S12.1 Fracture of second cervical vertebra

S12.2 Fracture of other specified cervical vertebra

S13.1 Dislocation of cervical vertebra

S14.1 Other and unspecified injuries of cervical spinal cord

S24.1 Other and unspecified injuries of thoracic spinal cord

S34.1 Other injury of lumbar spinal cord

T85.6 Mechanical complication of other specified internal prosthetic devices, implants and grafts

Patients with the following diagnosis were categorized into the **others** group:

A17.0† Tuberculous meningitis

A17.8† Other tuberculosis of nervous system

A41.9 Sepsis, unspecified

A83.0 Japanese encephalitis

A86 Unspecified viral encephalitis

B67.6 Echinococcus multilocularis infection, other and multiple sites

B67.9 Echinococcosis, other and unspecified

B89 Unspecified parasitic disease

G00.9 Bacterial meningitis, unspecified

G04.2 Bacterial meningoencephalitis and meningomyelitis, not elsewhere classified

G04.9 Encephalitis, myelitis and encephalomyelitis, unspecified

G06.0 Intracranial abscess and granuloma

G20 Parkinson disease

G24.9 Dystonia, unspecified (Dyskinesia NOS)

G40.5 Special epileptic syndromes

G40.9 Epilepsy, unspecified

G50.0 Trigeminal neuralgia

G91.0 Communicating hydrocephalus

G91.1 Obstructive hydrocephalus

G91.3 Post-traumatic hydrocephalus, unspecified

G91.9 Hydrocephalus, unspecified

G93.2 Benign intracranial hypertension

G96.0 Cerebrospinal fluid leak

G97.2 Intracranial hypotension following ventricular shunting

G97.8 Other postprocedural disorders of nervous system

J15.9 Bacterial pneumonia, unspecified

J18.9 Pneumonia, unspecified

J94.8 Other specified pleural conditions

J98.4 Other disorders of lung

Q03.9 Congenital hydrocephalus, unspecified

T81.0 Haemorrhage and haematoma complicating a procedure, not elsewhere classified

T81.4 Infection following a procedure, not elsewhere classified

T82.8 Other specified complications of cardiac and vascular prosthetic devices, implants and grafts

T84.4 Mechanical complication of other internal orthopaedic devices, implants and grafts

T85.0 Mechanical complication of ventricular intracranial (communicating) shunt

T85.7 Infection and inflammatory reaction due to other internal prosthetic devices, implants and grafts

T85.8 Other complications of internal prosthetic devices, implants and grafts, not elsewhere classified

Z09.0 Follow-up examination after surgery for other conditions

Z42.0 Follow-up care involving plastic surgery of head and neck

Z45.9 Adjustment and management of unspecified implanted device
